# Body mass index and stroke incidence in Japanese community residents: The Jichi Medical School (JMS) Cohort Study

**DOI:** 10.1016/j.je.2016.08.007

**Published:** 2017-03-07

**Authors:** Nami Kawate, Kazunori Kayaba, Motohiko Hara, Kazuhiko Kotani, Shizukiyo Ishikawa, the Jichi Medical School Cohort Study Group

**Affiliations:** a Graduate School of Saitama Prefectural University, Koshigaya, Saitama, Japan; b Department of Clinical Laboratory Medicine, Department of Public Health, Jichi Medical University, Shimotsuke, Tochigi, Japan; c Division of Community and Family Medicine, Center for Community Medicine, Jichi Medical University, Shimotsuke, Tochigi, Japan

**Keywords:** Body mass index, Community-based cohort study, Stroke, Japanese population, Sex difference

## Abstract

**Background:**

High body mass index (BMI) has been reported as a risk factor for cardiovascular events in Western countries, while low BMI has been reported as a risk factor for cardiovascular death in Asian countries, including Japan. Although stroke is a major cause of death and disability in Japan, few cohort studies have examined the association between BMI and stroke incidence in Japan. This study aimed to examine the association between BMI and stroke incidence using prospective data from Japanese community residents.

**Methods:**

Data were analyzed from 12,490 participants in the Jichi Medical School Cohort Study. Participants were categorized into five BMI groups: ≤18.5, 18.6–21.9, 22.0–24.9, 25.0–29.9, and ≥30.0 kg/m^2^. Multivariate-adjusted hazard ratios (HRs) and 95% confidence intervals (CIs) were calculated using the Cox proportional hazard model. The group with a BMI of 22.0–24.9 kg/m^2^ was used as the reference category.

**Results:**

During mean follow-up of 10.8 years, 395 participants (207 men and 188 women) experienced stroke, including 249 cerebral infarctions and 92 cerebral hemorrhages. Men with a BMI ≤18.5 kg/m^2^ (HR 2.11; 95% CI, 1.17–3.82) and women with a BMI ≥30.0 kg/m^2^ (HR 2.25; 95% CI, 1.28–5.08) were at significantly higher risk for all-stroke. Men with a BMI ≤18.5 kg/m^2^ were at significantly higher risk for cerebral infarction (HR 2.15; 95% CI, 1.07–4.33).

**Conclusions:**

The association between BMI and stroke incidence observed in this population was different than those previously reported: low BMI was a risk factor for all-stroke and cerebral infarction in men, while high BMI was a risk factor for all-stroke in women.

## Highlights

• The association of body mass index (BMI) with stroke remains controversial in Japan.
• Our large cohort study examined the association of interest in a Japanese population.
• Low BMI increased the risk of all-stroke and cerebral infarction incidence in men.
• In contrast, high BMI increased the risk of all-stroke incidence in women.

## Introduction

Stroke is a major cause of death and disability in Japan. In 2013, stroke was responsible for nearly 120,000 deaths, and stroke currently accounts for 9.3 percent of all-cause death in Japan.^1^ Body mass index (BMI) is used as a measure of body fat metabolism and has been used to define obesity, overweight, and leanness in numerous epidemiological studies.^2^ Epidemiologic studies^3^, ^4^, ^5^ in Western and Asian countries have reported that high BMI is a significant risk factor for mortality due to cardiovascular disease (CVD), including stroke. However, several epidemiologic studies on the association between BMI and stroke mortality in Japan have reported non-linear relationships^6^, ^7^, ^8^, ^9^, ^10^, ^11^ and no significant association.^12^

Considering the serious consequences of stroke, information regarding stroke incidence and mortality is important. Among the Japanese population, the acute case-fatality rate for cerebral infarction is less than 10 percent.^13^, ^14^ Nevertheless, non-fatal stroke is a major cause of lifelong disability and places a heavy burden on Japan's long-term care insurance system.^15^

European and North American studies^16^, ^17^, ^18^, ^19^, ^20^, ^21^, ^22^ have reported that high BMI is significantly associated with an increased incidence of stroke. However, in some of those studies,^18^, ^22^ the significance of this association disappeared after adjusting for potential confounders.

To the best of our knowledge, only a few previous cohort studies^23^, ^24^, ^25^ have examined the association between BMI and stroke incidence in Japan. Additionally, the sex ratios, implementation periods, stroke types, BMI ranges, and adjusted factors differed among these studies; therefore, the association between BMI and stroke incidence in the Japanese population remains unclear.

In this study, we examined the association between BMI and stroke incidence in Japanese community residents using data from the Jichi Medical School Cohort Study.

## Methods

### Study population

We obtained baseline data from the Jichi Medical School Cohort Study, a population-based prospective study conducted in 12 rural Japanese communities between April 1992 and July 1995 that investigated risk factors for CVD.^26^ In accordance with the Health and Medical Service Law for the Aged of 1982, mass screening examinations for CVD have been conducted since 1983. Study data were collected on the basis of these examination results. The participants for the mass screening examinations were residents aged 40–69 years in eight areas, and residents aged 19 years and older in another area. Participants from other age groups in the remaining three areas were also included.

In each community, a local government office sent personal invitations to all participants by mail. Among the 12,490 individuals (4911 men and 7579 women) who participated in the mass screening examinations and underwent a basic medical checkup, the response rate was about 99%. Those who did not agree to be followed (n = 95), those without BMI data (n = 504), and/or those with a history of stroke (n = 113), myocardial infarction (n = 65), angina pectoris (n = 221) or cancer (n = 142) were excluded. Ultimately, overlapping data from 11,404 participants (4444 men and 6960 women; age range, 19–90 years) were analyzed.

### Baseline examinations

Health checkups were carried out in each community. Body height was measured without shoes, and body weight was recorded while fully clothed and then adjusted by subtracting 0.5 kg (in the summer) or 1 kg (in other seasons) to account for clothing. BMI was calculated as weight (kg) divided by the square of height (m). Systolic blood pressure (SBP) was measured using a fully automatic sphygmomanometer (BP203RV-II; Nippon Colin, Komaki, Japan). Serum lipids (total cholesterol [TC], high-density lipoprotein [HDL] cholesterol, and triglycerides [TG]) and blood glucose (BG) were also measured using standard methods, as previously reported.^26^

Information regarding medical history and sociodemographic characteristics was obtained by trained interviewers using standardized questionnaires. Smoking status was defined as current smoker, ex-smoker, or never smoker, and alcohol drinking status was categorized as current drinker, ex-drinker, or never drinker.

### Follow-up

We obtained baseline data from the mass screening examinations and subsequently attempted to follow all of the participants annually. We asked the participants directly whether they had experienced stroke or myocardial infarction after the baseline study. If they had, we asked which hospital they had visited and when they did so in order to ascertain the incidence of disease. The participants who had not undergone screening examinations were contacted via mail or telephone or visited at home by a public health nurse. We also checked medical records to verify whether the participants had visited the hospital. If an incident case was suspected, we collected computed tomography or magnetic resonance imaging for evidence of stroke, or electrocardiograms for evidence of myocardial infarction. Death certificates were collected at public health centers with permission from the Agency of General Affairs and the Ministry of Health, Labour and Welfare. Based on data obtained annually from each municipal government, a total of 386 participants moved out of the study area during the follow-up period. We stopped following such participants on the day they left their respective areas. Follow-up was also discontinued for participants who died before the end of the study. Death resulting from CVD was included in the CVD incidence data. Follow-up of all participants was continued until the end of 2005.

### Diagnostic criteria

CVD was defined as stroke, myocardial infarction, or sudden death, whichever occurred first. The diagnoses were determined independently by a diagnosis committee consisting of a radiologist, a neurologist, and two cardiologists. To establish diagnosis, stroke was defined as sudden onset of a focal, non-convulsive neurological deficit persisting longer than 24 h. Stroke subtypes were classified as cerebral infarction, hemorrhagic stroke (cerebral hemorrhage and subarachnoid hemorrhage), or undetermined according to the criteria of the National Institute of Neurological Disorders and Stroke.^27^ Myocardial infarction was diagnosed according to the criteria of the World Health Organization Multinational Monitoring of Trends and Determinants in Cardiovascular Disease Project.^28^ Details of the design of this study have been described previously.^26^

### Statistical analysis

All analyses were performed separately for men and women using SPSS for Windows (version 21.0; IBM Japan, Inc., Tokyo, Japan). First, BMI was categorized into the following five groups based partly on the Criteria for Obesity Disease by the Japan Society for the Study of Obesity: ≤18.5, 18.6–21.9, 22.0–24.9, 25.0–29.9, and ≥30.0 kg/m^2^.^29^ One-way analysis of variance and the chi-square test for variables were performed to clarify the associations between BMI and potential confounders. Finally, the Cox proportional hazards model was used to calculate hazard ratios (HRs) and 95% confidence intervals (CIs) for stroke incidence in relation to BMI, adjusting for age (HR1), as well as SBP, TC, HDL cholesterol, TG, diabetes mellitus (DM), smoking status and alcohol drinking status (HR2). All categorical variables, including BMI, were treated as dummy variables. The group with a BMI 22.0–24.9 was used as the reference category in all analyses. Age, SBP, TC, HDL cholesterol and TG were entered into the model as continuous variables. DM (fasting BG ≥ 126 mg/dL or casual BG ≥ 200 mg/dL, or history of use of diabetic medication), smoking status (current, ex-, or never smoker), and alcohol drinking status (current, ex-, or never drinker) were entered as categorical variables.

All reported P values are two-tailed. P values < 0.05 were considered statistically significant.

### Ethical considerations

This study was approved by the Institutional Review Board of Jichi Medical School (Epidemiology 03-01) and the Ethics Committee of Saitama Prefectural University (25518). Written informed consent was obtained from all participants.

## Results

The baseline characteristics of participants by BMI group are shown in Table 1. In both sexes, BMI was positively correlated with SBP, TC, and TG, and inversely correlated with HDL cholesterol. The group with a BMI ≥30.0 kg/m^2^ tended to have DM, and men in the higher BMI groups were less likely to be current and ex-smokers.

**Table 1:** Baseline relationships between body mass index and potential confounders.

	Body mass index, kg/m^2^					P-value^a^	Body mass index, kg/m^2^					P-value^a^
	≤18.5	18.6–21.9	22.0–24.9	25.0–29.9	≥30.0		≤18.5	18.6–21.9	22.0–24.9	25.0–29.9	≥30.0	
	Men						Women					
Number of subjects	190	1533	1725	932	64		365	2272	2567	1569	187	
Age, years	59.6 (13.4)	55.3 (12.5)	54.9 (11.5)	53.3 (11.0)	53.3 (11.5)	<0.01	55.2 (14.6)	53.3 (12.2)	55.8 (10.2)	56.8 (9.6)	55.4 (9.1)	<0.01
Systolic blood pressure, mm Hg	123.6 (20.7)	126.6 (19.8)	132.1 (19.9)	138.2 (19.9)	145 (20.1)	<0.01	118.3 (20.5)	122.4 (19.9)	129.3 (20.1)	135.3 (20.5)	140.6 (21.5)	<0.01
Serum cholesterol concentration												
Total cholesterol, mg/dL	171.6 (30.8)	178.3 (33.2)	187.2 (33.0)	193.7 (34.8)	202.2 (36.9)	<0.01	186.4 (35.5)	191.5 (34.5)	197.9 (34.2)	204.5 (34.3)	206.1 (34.4)	<0.01
High-density lipoprotein cholesterol, mg/dL	55.3 (15.5)	52.4 (13.3)	48 (13.0)	43.8 (11.5)	40.9 (11.6)	<0.01	58.6 (13.3)	55.6 (12.4)	51.8 (12.2)	48.9 (11.3)	47.2 (10.9)	<0.01
Triglycerides, mg/dL	88.5 (58.4)	103.5 (72.7)	130.8 (80.1)	167.1 (101.3)	221.5 (150.5)	<0.01	83.0 (43.8)	91.6 (48.4)	111.8 (63.7)	135.7 (85.5)	150.5 (89.2)	<0.01
Diabetes mellitus,^b^ %	4.2	4.2	4.4	5.1	7.8	0.57	1.7	1.4	1.6	2.1	9.6	<0.01
Current smoker, %	62.2	57.4	47.2	44.8	45.8	<0.01	8.5	6.9	4.2	4.9	8.1	<0.01
Current alcohol drinker, %	65.2	76.4	76.9	74.9	56.6	<0.01	32.4	33.2	34.9	33.5	30.7	0.61

Data are expressed as mean (standard deviation) or percentage of participants.

a P values were calculated using one-way analysis of variance or the chi-square test for variables.

b Fasting blood glucose level ≥126 mg/dL or casual blood glucose level ≥200 mg/dL, or history of use of diabetic medication.

During an average follow-up period of 10.8 years, 395 participants (207 men and 188 women) experienced stroke. Regarding the type of stroke, 249 cerebral infarctions (149 men and 100 women), 92 cerebral hemorrhages (45 men and 47 women), and 54 subarachnoid hemorrhages (13 men and 41 women) were reported.

**Fig. 1:**
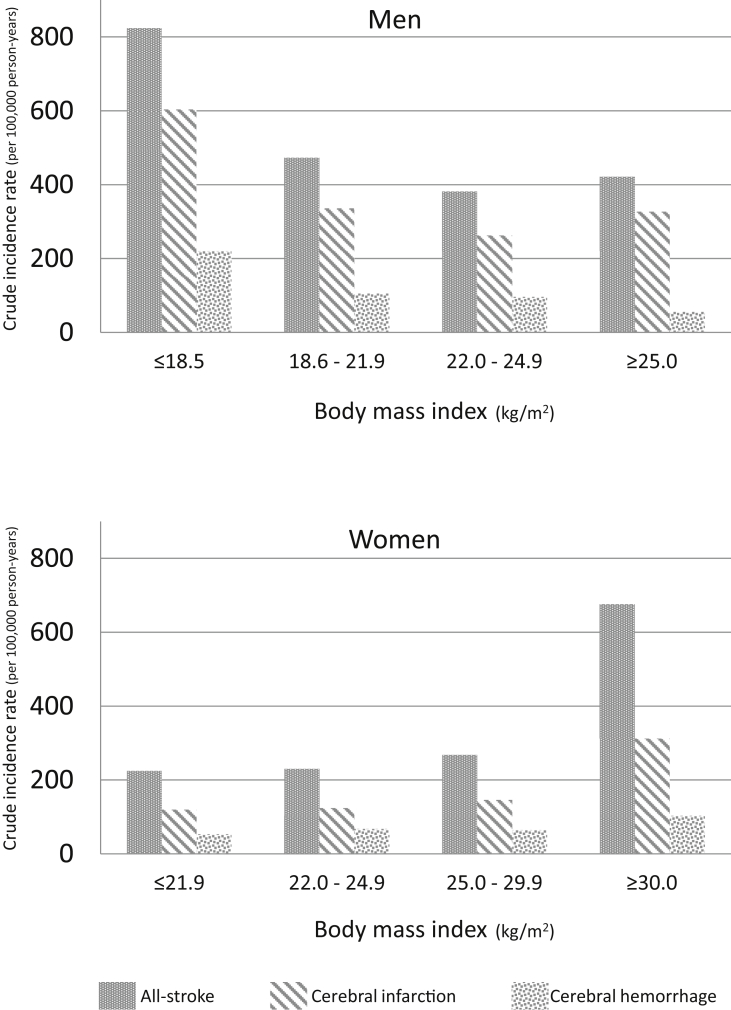
Crude incidence rate based on body mass index.

Incidence rates for stroke in each BMI group are shown in Table 2, Table 3 and in Fig. 1. In men, the group with a BMI ≤18.5 kg/m^2^ had the highest stroke incidence. Conversely, in women, the group with a BMI ≥30.0 kg/m^2^ had the highest stroke incidence.

**Table 2:** Hazard ratios based on body mass index and adjusted for potential confounders in men.

	Body mass index, kg/m^2^			
	≤18.5	18.6–21.9	22.0–24.9	≥25.0
Person-years	1820	16,092	18,613	10,676
All-stroke				
Number of cases	15	76	71	45
Incidence rate^a^	824	472	381	421
HR1 (95% CI)	1.52 (0.87–2.66)	1.16 (0.84–1.61)	1.00	1.31 (0.90–1.90)
HR2 (95% CI)	2.11 (1.17–3.82)	1.35 (0.95–1.91)	1.00	0.97 (0.64–1.48)
Cerebral infarction				
Number of cases	11	54	49	35
Incidence rate^a^	604	336	263	327
HR1 (95% CI)	1.59 (0.82–3.07)	1.19 (0.81–1.76)	1.00	1.51 (0.98–2.32)
HR2 (95% CI)	2.15 (1.07–4.33)	1.42 (0.94–2.15)	1.00	1.13 (0.69–1.83)
Cerebral hemorrhage				
Number of cases	4	17	18	6
Incidence rate^a^	220	106	97	56
HR1 (95% CI)	1.70 (0.57–5.07)	1.04 (0.54–2.02)	1.00	0.66 (0.26–1.65)
HR2 (95% CI)	2.78 (0.86–9.01)	1.11 (0.53–2.33)	1.00	0.43 (0.14–1.31)

CI, confidence interval; HR, hazard ratio.

HR1: Hazard ratios adjusted for age.

HR2: Hazard ratios adjusted for age, systolic blood pressure, total cholesterol, high-density lipoprotein cholesterol, triglycerides, diabetes mellitus, smoking, and alcohol consumption.

a Per 100,000 person-years.

**Table 3:** Hazard ratios based on body mass index and adjusted for potential confounders in women.

	Body mass index, kg/m^2^			
	≤21.9	22.0–24.9	25.0–29.9	≥30.0
Person-years	28,442	28,285	17,135	1924
All-stroke				
Number of cases	64	65	46	13
Incidence rate^a^	225	230	268	676
HR1 (95% CI)	1.03 (0.73–1.45)	1.00	1.15 (0.79–1.67)	3.61 (1.99–6.57)
HR2 (95% CI)	1.12 (0.78–1.60)	1.00	0.94 (0.62–1.41)	2.25 (1.28–5.08)
Cerebral infarction				
Number of cases	34	35	25	6
Incidence rate^a^	120	124	146	312
HR1 (95% CI)	1.24 (0.86–1.80)	1.00	1.52 (0.98–2.36)	1.36 (0.32–5.57)
HR2 (95% CI)	1.03 (0.63–1.70)	1.00	0.90 (0.51–1.59)	2.48 (0.94–6.56)
Cerebral hemorrhage				
Number of cases	15	19	11	2
Incidence rate^a^	53	67	64	103
HR1 (95% CI)	0.82 (0.41–1.61)	1.00	0.94 (0.45–1.97)	1.95 (0.45–8.43)
HR2 (95% CI)	0.94 (0.46–1.94)	1.00	0.88 (0.40–1.94)	2.41 (0.54–10.72)

CI, confidence interval; HR, hazard ratio.

HR1: Hazard ratios adjusted for age.

HR2: Hazard ratios adjusted for age, systolic blood pressure, total cholesterol, high-density lipoprotein cholesterol, triglycerides, diabetes mellitus, smoking, and alcohol consumption.

a Per 100,000 person-years.

Adjusted HRs and 95% CIs are shown also in Table 2, Table 3. In men, the BMI 25.0–29.9 kg/m^2^ group was combined with the BMI ≥30.0 kg/m^2^ group because both groups only had one case of cerebral infarction, and only the BMI ≥30.0 kg/m^2^ group had a case of hemorrhagic stroke. In women, the BMI ≤18.5 group was combined with the BMI 18.6–21.9 kg/m^2^ group because no cases of hemorrhagic stroke were reported in the BMI ≤18.5 group.

In men, the HR2 for all-stroke was significantly higher in the BMI ≤18.5 kg/m^2^ group (HR2 2.11; 95% CI, 1.17–3.82). In women, the BMI ≥30.0 kg/m^2^ group had significantly higher HR1 (HR1 3.61; 95% CI, 1.99–6.57) and HR2 (HR2 2.25; 95% CI, 1.28–5.08) for all-stroke. HR2 for cerebral infarction was high with borderline significance (HR2 2.48; 95% CI, 0.94–6.56). HR2 for cerebral hemorrhage was also high, but not statistically significant (HR2 2.41; 95% CI, 0.54–10.72). In addition, HRs were calculated using BMI as a continuous value. HR1 for all-stroke in women and HR2 for cerebral hemorrhage in men were statistically significant (eTable 1).

## Discussion

After adjusting for potential confounders, men with a BMI ≤18.5 kg/m^2^ were at significantly higher risk for all-stroke and cerebral infarction, whereas women with a BMI ≥30.0 kg/m^2^ were at increased risk for all-stroke.

To the best of our knowledge, the association between BMI and stroke incidence in Japan has only been evaluated in three previous cohort studies. Although our study was similar to those previous studies in terms of design, period of implementation, and age range of participants, our results were inconsistent with previous findings. The Hisayama study reported that high BMI was associated with a high incidence of cerebral infarction in men, but not in women.^25^ The Japan Public Health Center-Based Prospective (JPHC) Study reported that higher BMI was associated with an increased risk of stroke in women, but not in men.^23^ Females in the Hisayama study were more likely to be current smokers than those in our study. In addition, the JPHC study used a self-administered questionnaire to identify hypertension, DM, and dyslipidemia, and calculated BMI using self-reported information. These differences could explain the inconsistencies in the findings between our study and the previous studies. A meta-analysis performed by the Japan Atherosclerosis Longitudinal Study group reported a significantly elevated incidence of cerebral infarction and hemorrhage in both sexes with a BMI ≥27.5 kg/m^2^, although this significance disappeared after adjusting for SBP.^24^ In our study, adjusting for SBP did not attenuate the significance of the association between high BMI and an elevated risk of stroke in women.

Significant positive associations have been reported between high BMI and incidence of cerebral infarction in both sexes in North American and European studies.^16^, ^17^, ^18^, ^19^, ^20^, ^21^, ^22^ However, some of those results were no longer statistically significant after adjusting for potential confounders, such as hypertension, DM, and dyslipidemia.^18^, ^22^ In addition, to date, no significant association between low BMI and an increased risk of stroke incidence has been reported. Therefore, our study may represent new epidemiologic findings.

Furthermore, in terms of studies in which the end point was mortality, only non-linear relationships, such as U-shaped,^7^, ^10^, ^11^ J-shaped,^6^ and inverted J-shaped relationships,^8^ have been reported between BMI and stroke mortality. Our incidence study observed a similar relationship to that reported in the Miyako study (conducted in Japan), in which low BMI appeared to be associated with an increased risk of stroke mortality in men.^9^

Our results suggest that low BMI in men is associated with an increased risk of stroke. Even though BMI measurement is limited in that fat-related weight is not distinguished from muscle-related weight, it is widely used and well accepted in epidemiologic research and clinical practice. Although the association between physiological factors, such as obesity, leanness, and proneness to disease, remains unknown, low BMI may result in health risks, such as low muscle mass, which are associated with worsening nutritional status, poor physical fitness, inflammation, and altered hormonal milieu.^30^ Further investigations are needed to achieve a better understanding of the risk of stroke in patients with low BMI.

The primary strength of our study was that it evaluated stroke incidence among both sexes based on a large Japanese cohort study. In addition, data were obtained in a standardized fashion. Only validated cases of stroke among annual health examination participants who had no history of CVD at baseline were included. The diagnosis of stroke was made by an independent committee using accepted diagnostic criteria, which minimized the possibility of information bias.

However, this study did have several limitations. First, although the study participants were selected from a population-based health examination, the selections were not randomized. Among the health examination participants, the proportions treated for hypertension, DM, and/or dyslipidemia were lower than those reported in a national health and nutrition examination survey.^31^ Therefore, the participants in this study appeared to be somewhat healthier than the general population. Second, smoking status, alcohol drinking status, and history of medication were all self-reported, and the participants were weighed with their clothes on; therefore, some inaccuracies can be expected. Third, cases involving asymptomatic stroke were not included; therefore, stroke incidence may have been underestimated. Finally, the significant findings were based on 11 incident stroke cases involving lean men and 13 cases involving obese women. Even though these results were statistically significant after adjusting for age and other major potential CVD risk factors, these findings may have been a chance observation.

## Conclusion

The results of this study suggest that men with a BMI ≤18.5 kg/m^2^ and women with a BMI ≥30.0 kg/m^2^ are at significantly higher risk for all-stroke, and men with a BMI ≤18.5 kg/m^2^ are at significantly increased risk for cerebral infarction. These results could provide potentially useful information to stimulate further studies regarding the association between BMI and stroke incidence among Japanese community residents.

## Financial disclosure

The authors have no financial relationships to disclose.

## Conflicts of interest

None declared.

## Appendix A Supplementary data

eTable 1 Hazard ratios for stroke incidence when body mass index is used as a continuous variable.
