# Supplementary material for: Body mass index and stroke incidence in Japanese community residents: The Jichi Medical School (JMS) Cohort Study
Source: J Epidemiol. 2017 Mar 7;27(7):325–30. doi: 10.1016/j.je.2016.08.007 (PMC5498403; doi:10.1016/j.je.2016.08.007)
Supplement: eTable 1 — Hazard ratios for stroke incidence when body mass index is used as a continuous variable. [file mmc1.pdf]

**eTable 1.** Hazard ratios for stroke incidence when body mass index is used as a continuous variable

| | Men | Women |
|---|---|---|
| Person-years | 40,940 | 65,983 |
| All-stroke | | |
| Number of cases | 181 | 172 |
| Incidence rate[a] | 442 | 260 |
| HR1 (95% CI) | 1.01 ( 0.98 - 1.07 ) | 1.05 ( 1.01 - 1.10 ) |
| HR2 (95% CI) | 0.96 ( 0.90 - 1.01 ) | 1.01 ( 0.96 - 1.07 ) |
| Cerebral infarction | | |
| Number of cases | 129 | 91 |
| Incidence rate[a] | 315 | 137 |
| HR1 (95% CI) | 1.04 ( 0.98 - 1.10 ) | 1.06 ( 0.99 - 1.12 ) |
| HR2 (95% CI) | 0.97 ( 0.91 - 1.04 ) | 1.02 ( 0.95 - 1.09 ) |
| Cerebral hemorrhage | | |
| Number of cases | 39 | 43 |
| Incidence rate[a] | 95 | 65 |
| HR1 (95% CI) | 0.95 ( 0.85 - 1.06 ) | 1.06 ( 0.98 - 1.16 ) |
| HR2 (95% CI) | 0.86 ( 0.75 - 0.99 ) | 1.06 ( 0.95 - 1.17 ) |

CI, confidence interval; HR, hazard ratio.

HR1: Hazard ratios adjusted for age.

HR2: Hazard ratios adjusted for age, systolic blood pressure, total cholesterol, high-density lipoprotein cholesterol, triglycerides, diabetes mellitus, smoking, and alcohol consumption.

[a]per 100,000 person-years
